# Perioperative red blood cell transfusion is associated with poor functional outcome and overall survival in patients with newly diagnosed glioblastoma

**DOI:** 10.1007/s10143-021-01633-y

**Published:** 2021-09-04

**Authors:** Matthias Schneider, Niklas Schäfer, Anna-Laura Potthoff, Leonie Weinhold, Lars Eichhorn, Johannes Weller, Elisa Scharnböck, Christina Schaub, Muriel Heimann, Erdem Güresir, Felix Lehmann, Hartmut Vatter, Ulrich Herrlinger, Patrick Schuss

**Affiliations:** 1 Department of Neurosurgery, University Hospital Bonn, Venusberg-Campus 1, 53127 Bonn, Germany; 2 Division of Clinical Neuro-Oncology, Department of Neurology, University Hospital Bonn, Bonn, Germany; 3 Institute of Medical Biometry, Informatics and Epidemiology (IMBIE), University Hospital Bonn, Bonn, Germany; 4 Department of Anesthesiology and Critical Care Medicine, University Hospital Bonn, Bonn, Germany

**Keywords:** Perioperative blood transfusion, RBC, Survival, Glioblastoma, Cancer, Brain tumor

## Abstract

The influence of perioperative red blood cell (RBC) transfusion on prognosis of glioblastoma patients continues to be inconclusive. The aim of the present study was to evaluate the association between perioperative blood transfusion (PBT) and overall survival (OS) in patients with newly diagnosed glioblastoma. Between 2013 and 2018, 240 patients with newly diagnosed glioblastoma underwent surgical resection of intracerebral mass lesion at the authors’ institution. PBT was defined as the transfusion of RBC within 5 days from the day of surgery. The impact of PBT on overall survival was assessed using Kaplan–Meier analysis and multivariate regression analysis. Seventeen out of 240 patients (7%) with newly diagnosed glioblastoma received PBT. The overall median number of blood units transfused was 2 (95% CI 1–6). Patients who received PBT achieved a poorer median OS compared to patients without PBT (7 versus 18 months; *p* < 0.0001). Multivariate analysis identified “age > 65 years” (*p* < 0.0001, OR 6.4, 95% CI 3.3–12.3), “STR” (*p* = 0.001, OR 3.2, 95% CI 1.6–6.1), “unmethylated MGMT status” (*p* < 0.001, OR 3.3, 95% CI 1.7–6.4), and “perioperative RBC transfusion” (*p* = 0.01, OR 6.0, 95% CI 1.5–23.4) as significantly and independently associated with 1-year mortality. Perioperative RBC transfusion compromises survival in patients with glioblastoma indicating the need to minimize the use of transfusions at the time of surgery. Obeying evidence-based transfusion guidelines provides an opportunity to reduce transfusion rates in this population with a potentially positive effect on survival.

**Supplementary Information:**

The online version contains supplementary material available at 10.1007/s10143-021-01633-y.

## Introduction

Perioperative anemia requires the transfusion of red blood cells (RBC) after reaching an individual threshold value, which often varies considerably for interhospital comparison. Due to the manifold effects of perioperative blood transfusion (PBT) on morbidity and mortality, opinions differ on the effect of PBT on the overall survival (OS) of cancer patients. Some studies reported an association between PBT and poorer OS, while others could not demonstrate this association [14, 15, 24].

Especially in the context of (semi-)elective intracranial surgical procedures, current efforts in patient blood management are aimed at raising the thresholds for transfusions while maintaining patient safety, leading to a controversy between restrictive and liberal transfusion strategies [11]. Perioperative anemia with concomitant oxygen starvation and thus endangerment of important organ functions must be assessed individually against a potentially harmful interaction or reactions of the blood transfusions that need to be carried out [1, 16]. For this, daily perioperative and individual risk and benefit analyses are essential to understand the consequences of PBT in the respective patient cohorts. In the case of patients suffering from glioblastoma, the available data is very scarce.

In the present study, we aimed at assessing a potential correlation of PBT and further pre- and intraoperatively collectable variables with impaired functional outcome and worsened overall survival in patients that had undergone neurosurgical resection of newly diagnosed glioblastoma at our institution.

## Methods

### Patients

Newly diagnosed glioblastoma patients who underwent surgical resection at the authors’ institution between 2013 and 2018 were entered into a computerized database. Only those patients with completely available preclinical, histopathological, and perioperative data were included in further analysis. The approval for this study was obtained from the institutional ethics committee. Information, including patient characteristics, radiological features, methylation status of the MGMT promoter, age-adjusted Charlson Comorbidity Index (CCI), functional neurological status and laboratory values at admission and during treatment, and the need for peri- and/or intraoperative blood transfusion, were collected and further analyzed. MGMT promotor methylation status was determined by the local department of neuropathology by pyrosequencing as published in 2007 [17]. The Karnofsky Performance Score (KPS) was utilized to assess patients according to their functional status as previously described [6, 21, 22]. In this context, KPS ≥ 70 was defined as a favorable outcome at postoperative follow-up assessments.

During the weekly interdisciplinary tumor advisory board meetings of the neuro-oncological center, individual treatment decisions were made in interdisciplinary consensus at the initial presentation of the patient and during follow-up.

The extent of resection (EOR) was evaluated in early (< 72 h) postoperative 3-T magnetic resonance imaging (MRI). Gross total resection (GTR) was defined as the complete removal of contrast-enhancing tissue. Preoperative tumor volumes volumetrically were assessed based on gadolinium-enhanced T1-weighted MRI using commercially available software (TumorTracking Tool, IntelliSpace Portal 5.0, Philips, the Netherlands). In accordance to Flores et al. [19], a cutoff value of 60 mL of tumor volume was chosen in order to analyze for a potential correlation between tumor volume and PBT. Every neurosurgeon in charge fulfilled the requirements for Neuro-Oncology Centers certificated by the German Cancer Society.

Perioperative blood transfusions were determined as any allogeneic transfusion of red blood cells (RBC) during or within 5 days after surgery for glioblastoma. The threshold values for RBC transfusions in clinically stable patients were specified at a hemoglobin (Hb) concentration of < 7 g/dL, following the current consensus recommendations of patient blood management [18].

Overall survival (OS) was measured from the day of glioblastoma surgery until death or last observation. All parameters were compared in terms of OS.

### Statistics

Data analysis was performed using the computer software packages SPSS (version 25, IBM Corp., Arminian, NY) and GraphPad Prism (version 8, GraphPad Software, La Jolla, CA). Unpaired categorical and binary variables were analyzed in contingency tables using the Fisher exact test. The Mann–Whitney *U*-test was chosen to compare continuous variables as data were not normally distributed. OS was analyzed using the Kaplan–Meier method and Gehan-Breslow-Wilcoxon test. Results with *p* < 0.05 were considered statistically significant. Furthermore, a backward stepwise method was used to construct a multivariate logistic regression model in order to find independent predictors of 1-year mortality in patients with glioblastoma who underwent surgical resection.

## Results

### Patient characteristics

Between 2013 and 2018, a total of 240 patients with newly diagnosed glioblastoma underwent surgery at the authors’ institution. Mean age of patients with glioblastoma was 62 ± 13 years. Regarding EOR, GTR was achieved in 164 patients (68%), while subtotal resection (STR) was achieved in 76 patients (32%). Twelve patients suffered from multifocal manifestation at time of surgery (5%). Median OS for patients with glioblastoma in the current patient cohort was 16 months (95% CI 14–18) (Table 1).

**Table 1:** Patient characteristics

	Glioblastoma patients without PBT (*n* = 223)	Glioblastoma patients with PBT (*n* = 17)	*p*-value
Mean age (± SD, yrs)	61 ± 13	71 ± 10	*p* = 0.001
Baseline Hb ≤ 13 (g/dL)	11 (5%)	4 (24%)	n.s
Baseline KPS ≥ 70	212 (95%)	15 (88%)	n.s
Age-adjusted CCI ≥ 5	35 (16%)	7 (41%)	*p* = 0.02
30-d mortality	5 (2%)	3 (18%)	*p* = 0.01
1-yr mortality	77 (35%)	13 (77%)	*p* = 0.001
KPS ≥ 70 after 3 mos	177 (79%)	8 (47%)	*p* = 0.0007
KPS ≥ 70 after 6 mos	157 (70%)	6 (35%)	*p* = 0.0008
KPS ≥ 70 after 1 yr	116 (52%)	0 (0%)	*p* < 0.0001
GTR	153 (69%)	11 (65%)	n.s
ASA < 3	162 (74%)	9 (53%)	n.s
Baseline CRP ≥ 5	33 (15%)	2 (12%)	n.s
Baseline WBC > 12	92 (41%)	9 (53%)	n.s
Operation time (± SD, min)	239 ± 78	283 ± 102	n.s
Mean blood loss (± SD, mL)	266 ± 255	900 ± 636	*p* < 0.0001
Intraop RBC transfusion	n.a	10 (59%)	n.a
Unmethylated MGMT status	120 (55%)	8 (47%)	n.s

*ASA*, American Association of Anesthesiologists; *CCI*, Charlson Comorbidity Index; *CRP*, C-reactive protein; *d*, day; *GTR*, gross total resection; *Hb*, hemoglobin; *KPS*, Karnofsky Performance Scale; *mons*, months; *PBT*, perioperative blood transfusion; *RBC*, red blood cell; *WBC*, white blood cell; *yr(s)*, year(s)

### Necessity of perioperative RBC transfusion

Overall, 17 patients required PBT (7%). The overall median unit of blood transfused was 2 units (95% CI 1–6). Of 17 patients, 2 received 1 unit (12%), 9 received 2 units (53%), and 6 received more than 2 units (35%). The mean age of patients who received PBT was 71 ± 10 years at the time of surgery. Therefore, patients with PBT were significantly older compared to patients without PBT (*p* = 0.002, 95% CI 3.5–15.8) (Table 1). Furthermore, the comorbidity burden on admission was significantly higher in patients with the necessity of PBT compared to patients without, according to the age-adjusted CCI (mean CCI 4.1 ± 2 versus CCI 2.6 ± 2.1, *p* = 0.005, 95% CI 0.39–2.52). Patients with the necessity of PBT suffered from significantly increased intraoperative blood loss (mean 900 ± 616 mL) compared to patients without PBT (mean 266 ± 255 mL; *p* < 0.0001, 95% CI 483–785). Nevertheless, admission Hb concentration did not differ between both groups (*p* = 0.24). Median blood loss in patients with preoperative anticoagulant medication was 200 mL (IQR 100–305) compared to 200 mL (IQR 100–390) in patients without preoperative anticoagulant medication (*p* = 0.75). Preoperative intake of anticoagulant medication did not significantly correlate to the extent of PBT: 5 out of 17 patients (29%) with PBT exhibited preoperative anticoagulant intake compared to 33 out of 223 patients (15%) without need for perioperative PBT (*p* = 0.16). Table 1 summarizes patient characteristics for patients who underwent PBT compared to patients who did not receive PBT.

Median tumor volume was 35 mL (IQR 12–72). The receiver operating characteristics (ROC) curve resulted in an area-under-the-curve value of 67.9 mL. Based on equal weighting of sensitivity and specificity, the cutoff of 125 mL was found to be the most efficient to separate between the two PBT groups. This cutoff leads to a sensitivity of 50.0% and a specificity of 94.3%. Eight out of 17 patients (47%) with PBT exhibited a tumor volume of ≥ 125 mL compared to 10 out of 223 patients (4%) without PBT (*p* < 0.001). The PBT group significantly more often suffered from postoperative LAE and pneumonia: 2 out of 17 patients (12%) within the PBT group suffered from pulmonary embolism during the postoperative hospital stay compared to 3 out of 223 patients (1%) within the non-PBT group (*p* = 0.04). Four out of 17 patients (24%) within the PBT group suffered from pneumonia during the postoperative hospital stay compared to 7 out of 223 patients (3%) within the non-PBT group (*p* = 0.004). Postoperative intensified treatment did not significantly differ for the PBT and the non-PBT group: 1 out of 17 patients (6%) with PBT received postoperative treatment according to the CeTeG protocol compared to 16 out of 207 patients (8%) without PBT (*p* = 1.0). TTF treatment was present in 0 out of 17 patients (0%) with PBT compared to 9 out of 214 patients (4%) without PBT (*p* = 1.0). For detailed information regarding the PBT group, see Supplementary Table S1.

### Influence of perioperative RBC transfusion on functional outcome

**Fig. 1:**
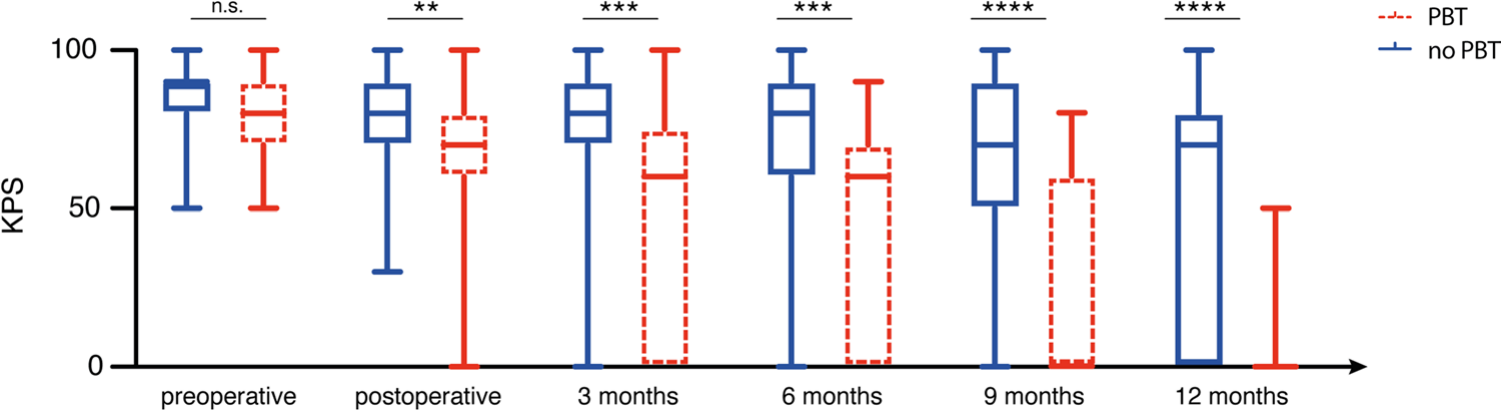
Perioperative RBC transfusion is associated with impaired postoperative functional outcome. Box and whisker plots depict KPS of patients with and without PBT pre- and postoperatively and at the 3-, 6-, 9-, and 12-month follow-up. KPS, Karnofsky Performance Scale; PBT, perioperative blood transfusion

Patients who received PBT achieved significantly less often favorable functional outcome compared to patients without perioperative need for RBC transfusion at follow-ups 3 months (*p* = 0.005, OR 3.8, 95% CI 1.5–9.3), 6 months (*p* = 0.005, OR 3.9, 9% CI 1.5–10.1), and 12 months (*p* < 0.0001, OR 37.9, 95% CI 2.3–638.9) postoperatively (Fig. 1, Table 1).

### Influence of perioperative RBC transfusion on survival

**Fig. 2:**
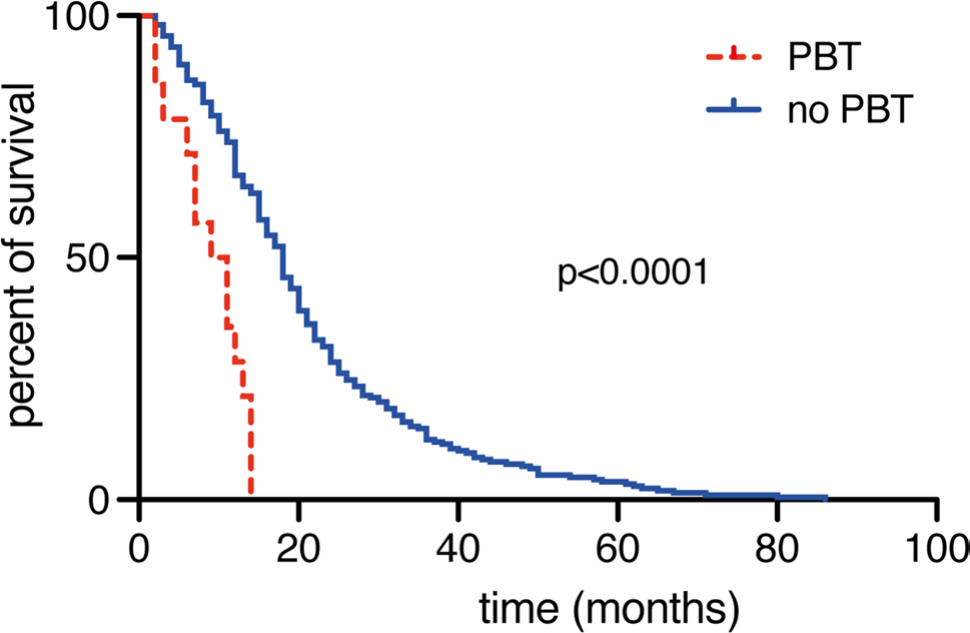
Perioperative RBC transfusion is associated with decreased overall survival rates. Kaplan–Meier curves for OS stratified into glioblastoma patients with and without PBT. OS, overall survival; PBT, perioperative blood transfusion

Overall survival in patients who received PBT was 7 months (95% CI 3–11). In patients who received PBT, median overall survival was significantly reduced compared to those who did not receive PBT (7 versus 18 months, *p* < 0.0001). Figure 2 presents Kaplan–Meier survival estimates categorized by transfusion status. Patients aged ≥ 70 years who received PBT exhibited a mOS of 6 months (IQR 2–11) compared to 12 months (7–18) for the geriatric patients without PBT (*p* = 0.01).

### Multivariate analysis

Multivariate analysis identified “age > 65 years” (*p* < 0.0001, OR 6.4, 95% CI 3.3–12.3), “STR” (*p* = 0.001, OR 3.2, 95% CI 1.6–6.1), “unmethylated MGMT status” (*p* < 0.001, OR 3.3, 95% CI 1.7–6.4), and “perioperative RBC transfusion” (*p* = 0.01, OR 6.0, 95% CI 1.5–23.4) as significantly and independently associated with 1-year mortality, whereas tumor volume did not in patients who had undergone glioblastoma surgery (Nagelkerke’s *R*^2^ 0.37).

## Discussion

For various reasons, blood loss might be considerable during oncosurgical cranial procedures despite the partly (semi-)elective character of these procedures. This may require transfusion of allogeneic red blood cells to maintain tissue perfusion and oxygen supply. Increasingly, it has been stated in the literature that perioperative transfusion of RBCs might lead to a reduced overall survival for different types of cancer [9, 12, 23]. However, data on the impact of perioperative RBC transfusion on overall survival in patients with newly diagnosed glioblastoma is scarce. The present study demonstrates that perioperative RBC transfusion in patients with newly diagnosed glioblastoma constitutes a significant and independent predictor of poor survival and correlates to worsened functional postoperative outcome.

Transfusion-related immunomodulation (TRIM) is considered to be the leading factor for poor oncological outcomes related to transfusion [9]. Various studies suggest an immunosuppressive effect of RBC transfusion and have indicated that TRIM appears to be largely due to the presence of donor white blood cell (WBC) populations in the transfused RBCs, which then negatively affect the immune system of the recipient [8]. However, with regard to potential cancer-promoting effects of RBC transfusions, aged erythrocytes rather than leukocytes, soluble factors, or storage time are also up for debate [2].

Besides eventual immunomodulatory aspects, the necessity of PBT should also consider the patient’s pre-existing comorbidity burden as well as the complexity and expected amount of blood loss within the procedure. Furthermore, elevated tumor volumes have been reported to correlate to higher intraoperative blood loss [13, 20] which might provide an additional rationale for PBT in patients with larger tumor burden. In the present study, glioblastoma patients with a perioperative necessity of RBC transfusion suffered a significantly increased intraoperative blood loss. Thus, 59% of all PBT in the selected study cohort were performed in a direct intraoperative context. Therefore, it might be assumed that in the significantly older, morbid patients with elevated tumor bulk, who were additionally weakened by the intraoperatively increased blood loss, an optimization of oxygen supply through RBC transfusions was attempted. Indeed, in the present series, we could observe a trend of a higher comorbidity burden in the patient group who received PBT though significantly different levels were not achieved. Patient blood management (PBM), in terms of implementing evidence-based guidelines for the use of blood products, aims to achieve optimal results based on the risk and benefit ratio for the patient. Current trends in PBM encourage the use of more conservative transfusion thresholds with a hemoglobin concentration of 7–8 g/dL in most clinical situations [7, 18]. These more restrictive strategies allow for a significant reduction in blood consumption, resulting in lower patient exposure to allogeneic RBCs. The oncological literature favors rather more restrictive transfusion strategies, although in numerous studies, a threshold value of 7 g/dL seems to be acceptable [3, 5]. In the current study, a restrictive transfusion strategy with a threshold value of 7 g/dL has been used in accordance with the hospital’s transfusion recommendation. It is plausible that such restrictive transfusion regimes may lead to a higher rate of PBT in the patient cohort with preoperative hemoglobin levels just above the defined threshold as very slight intraoperative blood loss will result in falling below these threshold values. In line with this, we could see a trend of lower baseline hemoglobin in the PBT compared to the non-PBT group. Nevertheless, there are various efforts and indications to expand the restrictive transfusion strategies in a patient-centered manner [4, 16]. RBC transfusion represents the most common therapy option for perioperative anemia in oncological patients. The blood-sparing technique of autologous cell harvesting is often not utilized in oncological surgery as a precautionary measure to avoid (re)introduction of tumor cells into the bloodstream. However, this safety concept seems to be increasingly questioned in some areas of oncological surgery [10].

Nevertheless, the influence of perioperative RBC transfusion on the postoperative neurological outcome and overall survival has been evaluated as independent of potential confounding factors in the present multivariate analysis. While to the best of our knowledge this effect has now been demonstrated for the first time in glioblastoma patients, it is consistent with results from similar studies in other cancer entities [9, 12, 23].

### Limitations

The present study has several limitations. Data collection was conducted retrospectively and reflects only the experience of one center. Therefore, an additional risk arises in this selected, retrospectively evaluated patient group in order to underestimate the influence of either the patient’s comorbidity, the severity of the disease, the complexity of the surgery, different postoperative treatment modalities, or the cumulative blood loss in relation to the observed results. Nevertheless, the present study provides a first-time description of a potential association of perioperative RBC transfusion with outcome and survival in patients with glioblastoma and should spark the demand for larger, multicenter studies.

## Conclusions

The present study indicates perioperative RBC transfusion to adversely affect functional neurological outcome and overall survival in patients with glioblastoma. Further studies will be needed in order to corroborate a potential need to seek options to reduce transfusion use at the time of surgery. Following evidence-based transfusion guidelines might offer an opportunity to minimize transfusion rates, which could have a positive impact on outcome and overall survival in glioblastoma patients.

## Supplementary Information

Below is the link to the electronic supplementary material.

Supplementary file1 (DOCX 20 KB)

## Data Availability

Restrictions apply to the availability of these data due to privacy restrictions.

## References

[1] Alan N, Seicean A, Seicean S, Neuhauser D, Weil RJ (2014). Impact of preoperative anemia on outcomes in patients undergoing elective cranial surgery. J Neurosurg.

[2] Atzil S, Arad M, Glasner A, Abiri N, Avraham R, Greenfeld K, Rosenne E, Beilin B, Ben-Eliyahu S (2008). Blood transfusion promotes cancer progression: a critical role for aged erythrocytes. Anesthesiology.

[3] Ballo O, Fleckenstein P, Eladly F, Kreisel EM, Stratmann J, Seifried E, Muller M, Serve H, Bug G, Bonig H, Brandts CH, Finkelmeier F (2020). Reducing the red blood cell transfusion threshold from 8.0 g/dl to 7.0 g/dl in acute myeloid leukaemia patients undergoing induction chemotherapy reduces transfusion rates without adversely affecting patient outcome. Vox Sang.

[4] Bergamin FS, Almeida JP, Landoni G, Galas F, Fukushima JT, Fominskiy E, Park CHL, Osawa EA, Diz MPE, Oliveira GQ, Franco RA, Nakamura RE, Almeida EM, Abdala E, Freire MP, Filho RK, Auler JOC, Hajjar LA (2017). Liberal versus restrictive transfusion strategy in critically ill oncologic patients: the transfusion requirements in critically ill oncologic patients randomized controlled trial. Crit Care Med.

[5] Boone JD, Kim KH, Marques M, Straughn JM (2014). Compliance rates and outcomes associated with a restrictive transfusion policy in gynecologic oncology patients. Gynecol Oncol.

[6] Buccheri G, Ferrigno D, Tamburini M (1996). Karnofsky and ECOG performance status scoring in lung cancer: a prospective, longitudinal study of 536 patients from a single institution. Eur J Cancer.

[7] Carson JL, Stanworth SJ, Roubinian N, Fergusson DA, Triulzi D, Doree C, Hebert PC (2016). Transfusion thresholds and other strategies for guiding allogeneic red blood cell transfusion. Cochrane Database Syst Rev.

[8] Cata JP, Wang H, Gottumukkala V, Reuben J, Sessler DI (2013). Inflammatory response, immunosuppression, and cancer recurrence after perioperative blood transfusions. Br J Anaesth.

[9] Connor JP, O’Shea A, McCool K, Sampene E, Barroilhet LM (2018). Peri-operative allogeneic blood transfusion is associated with poor overall survival in advanced epithelial ovarian cancer; potential impact of patient blood management on Cancer outcomes. Gynecol Oncol.

[10] Fischer D, Neb H, Choorapoikayil S, Zacharowski K, Meybohm P (2019). Red blood cell transfusion and its alternatives in oncologic surgery-a critical evaluation. Crit Rev Oncol Hematol.

[11] Gruenbaum SE, Ruskin KJ (2014). Red blood cell transfusion in neurosurgical patients. Curr Opin Anaesthesiol.

[12] Horowitz M, Neeman E, Sharon E, Ben-Eliyahu S (2015). Exploiting the critical perioperative period to improve long-term cancer outcomes. Nat Rev Clin Oncol.

[13] Kang Y, Wei KC, Toh CH (2019). Can we predict intraoperative blood loss in meningioma patients? Application of dynamic susceptibility contrast-enhanced magnetic resonance imaging. J Neuroradiol.

[14] Liu J, Chen S, Chen Y, Wang N, Ye X (2018). Perioperative blood transfusion has no effect on overall survival after esophageal resection for esophageal squamous cell carcinoma: a retrospective cohort study. Int J Surg.

[15] Liu X, Ma M, Huang H, Wang Y (2018). Effect of perioperative blood transfusion on prognosis of patients with gastric cancer: a retrospective analysis of a single center database. BMC Cancer.

[16] Meybohm P, Lindau S, Treskatsch S, Francis R, Spies C, Velten M, Wittmann M, Gueresir E, Stoppe C, Kowark A, Coburn M, Selleng S, Baschin M, Jenichen G, Meersch M, Ermert T, Zarbock A, Kranke P, Kredel M, Helf A, Laufenberg-Feldmann R, Ferner M, Wittenmeier E, Gurtler KH, Kienbaum P, de Abreu MG, Sander M, Bauer M, Seyfried T, Gruenewald M, Choorapoikayil S, Mueller MM, Seifried E, Brosteanu O, Bogatsch H, Hasenclever D, Zacharowski K, Group LC (2019). Liberal transfusion strategy to prevent mortality and anaemia-associated, ischaemic events in elderly non-cardiac surgical patients—the study design of the LIBERAL-Trial. Trials.

[17] Mikeska T, Bock C, El-Maarri O, Hubner A, Ehrentraut D, Schramm J, Felsberg J, Kahl P, Buttner R, Pietsch T, Waha A (2007). Optimization of quantitative MGMT promoter methylation analysis using pyrosequencing and combined bisulfite restriction analysis. J Mol Diagn.

[18] Mueller MM, Van Remoortel H, Meybohm P, Aranko K, Aubron C, Burger R, Carson JL, Cichutek K, De Buck E, Devine D, Fergusson D, Follea G, French C, Frey KP, Gammon R, Levy JH, Murphy MF, Ozier Y, Pavenski K, So-Osman C, Tiberghien P, Volmink J, Waters JH, Wood EM, Seifried E, Group IPF (2019). Patient blood management: recommendations from the 2018 Frankfurt Consensus Conference. JAMA.

[19] Palpan Flores A, Vivancos Sanchez C, Roda JM, Cerdan S, Barrios AJ, Utrilla C, Royo A, Gandia Gonzalez ML (2020). Assessment of pre-operative measurements of tumor size by MRI methods as survival predictors in wild type IDH glioblastoma. Front Oncol.

[20] Rajagopalan V, Chouhan RS, Pandia MP, Lamsal R, Rath GP (2019). Effect of intraoperative blood loss on perioperative complications and neurological outcome in adult patients undergoing elective brain tumor surgery. J Neurosci Rural Pract.

[21] Sacko A, Hou MM, Temgoua M, Alkhafaji A, Marantidou A, Belin C, Mandonnet E, Ursu R, Doridam J, Coman I, Levy-Piedbois C, Carpentier AF (2015). Evolution of the Karnosky Performance Status throughout life in glioblastoma patients. J Neurooncol.

[22] Schneider M, Potthoff AL, Keil VC, Güresir Á, Weller J, Borger V, Hamed M, Waha A, Vatter H, Güresir E, Herrlinger U, Schuss P (2019). Surgery for temporal glioblastoma: lobectomy outranks oncosurgical-based gross-total resection. J Neurooncol.

[23] Wang T, Luo L, Huang H, Yu J, Pan C, Cai X, Hu B, Yin X (2014). Perioperative blood transfusion is associated with worse clinical outcomes in resected lung cancer. Ann Thorac Surg.

[24] Warschkow R, Guller U, Koberle D, Muller SA, Steffen T, Thurnheer M, Schmied BM, Tarantino I (2014). Perioperative blood transfusions do not impact overall and disease-free survival after curative rectal cancer resection: a propensity score analysis. Ann Surg.

