# Supplementary material for: Perioperative red blood cell transfusion is associated with poor functional outcome and overall survival in patients with newly diagnosed glioblastoma
Source: Neurosurg Rev. 2021 Sep 4;45(2):1327–33. doi: 10.1007/s10143-021-01633-y (PMC8976811; doi:10.1007/s10143-021-01633-y)
Supplement: Supplementary file 1 — Supplementary file1 (DOCX 20 KB) [file 10143_2021_1633_MOESM1_ESM.docx]

**Supplementary Table S1:** Detailed patient characteristics for the PBT group

|  | **Sex** | **Age (ys)** | **Preop KPS** | **CCI** | **AC intake** | **Tumor volume** | **Intraop blood loss** | **RCC overall** | **KPS 3 months** | **Survival (months)** |
| --- | --- | --- | --- | --- | --- | --- | --- | --- | --- | --- |
| **Patient 1** | M | 57 | 100 | 3 | - | 147 | 600 | 2 | 70 | 14 |
| **Patient 2** | M | 76 | 70 | 5 | - | 71 | 1400 | 3 | 60 | 14 |
| **Patient 3** | M | 77 | 90 | 4 | - | 103 | 750 | 2 | 60 | 7 |
| **Patient 4** | F | 77 | 50 | 6 | - | 134 | 300 | 2 | 80 | 7 |
| **Patient 5** | M | 64 | 80 | 2 | - | 141 | 900 | 2 | 100 | 6 |
| **Patient 6** | M | 69 | 70 | 4 | - | 269 | 900 | 1 | 30 | 11 |
| **Patient 7** | M | 73 | 80 | 3 | - | 126 | 1600 | 2 | 70 | 12 |
| **Patient 8** | M | 59 | 70 | 2 | - | 212 | 900 | 6 | 0 | 0**^⁕^** |
| **Patient 9** | M | 53 | 70 | 1 | - | 200 | 2600 | 4 | 70 | 13 |
| **Patient 10** | F | 82 | 100 | 6 | - | 14 | 300 | 2 | 90 | 9 |
| **Patient 11** | M | 58 | 90 | 1 | - | 51 | 700 | 2 | 70 | 11 |
| **Patient 12** | M | 72 | 90 | 3 | - | 56 | 800 | 3 | 0 | 3 |
| **Patient 13** | M | 77 | 80 | 4 | + | 163 | 500 | 3 | 0 | 0^⁕⁕^ |
| **Patient 14** | F | 84 | 90 | 5 | + | 5 | 700 | 2 | 90 | 14 |
| **Patient 15** | M | 80 | 80 | 5 | + | 12 | 1700 | 2 | 0 | 0^⁕⁕^ |
| **Patient 16** | F | 67 | 90 | 8 | + | 5 | 150 | 6 | 0 | 2 |
| **Patient 17** | F | 78 | 60 | 7 | + | 7 | 500 | 1 | 0 | 2 |

⁕ death from cardiopulmonary failure within the 5^th^ week after surgery; ⁕⁕ death from secondary hemorrhage within the first week after surgery

AC, anticoagulation medication; CCI, Charlson comorbidity index adjusted for age; KPS, Karnofsky Performance Scale; PBT, perioperative blood transfusion; RCC, red cell concentrate; ys, years.
